# Improvements to visual working memory performance with practice and feedback

**DOI:** 10.1371/journal.pone.0203279

**Published:** 2018-08-30

**Authors:** Kirsten C. S. Adam, Edward K. Vogel

**Affiliations:** 1 Department of Psychology, University of Chicago, Chicago, Illinois, United States of America; 2 Institute for Mind and Biology, University of Chicago, Chicago, Illinois, United States of America; 3 Grossman Institute for Neuroscience, Quantitative Biology, and Human Behavior, University of Chicago, Chicago, Illinois, United States of America; Buenos Aires Physics Institute, ARGENTINA

## Abstract

Visual working memory capacity is estimated to be around 3–4 items, but on some trials participants fail to correctly report even a single item from the memory array. Such failures of working memory performance are surprisingly common, and participants have poor self-awareness of them. Previous work has shown that behavioral feedback can reduce the frequency of working memory failures, but the benefits of feedback disappeared immediately after it was taken away. Here, we tested whether extended practice with or without trial-by-trial feedback would lead to persistent improvements in working memory performance. Participants were assigned to one of four groups: (1) Working memory practice with feedback (2) Working memory practice without feedback (3) Crossword puzzle active control (4) No-contact control. Consistent with previous work, simple practice with a visual working memory task robustly improved working memory performance across practice sessions. However, we found only partial support for the efficacy of feedback in improving working memory performance. Practicing with feedback improved working memory performance relative to a no-feedback group for some practice sessions. However, the feedback benefits did not persist across all training sessions and did not transfer to a final test session without the feedback. Thus, the benefits of performance feedback did not persist over time. Further, we found only stimulus-specific transfer of visual working memory practice benefits. We also found that participants’ metaknowledge improved with practice, but that receiving feedback about task accuracy actually slightly harmed the accuracy of concurrent metaknowledge ratings. Finally, we discuss important design considerations for future work in this area (e.g. power, expectations, and “spacing effects”). For example, we found that achieved statistical power to detect a between-groups effect declined with practice. This finding has potentially critical implications for any study using a 1-session study to calculate power for a planned multi-session study.

## Introduction

Working memory is a key cognitive ability that helps us navigate the world around us. We use working memory to temporarily hold information in an active state and to manipulate it in aid of our current goals. Because of its importance for intelligent behaviors, there has great interest in improving working memory through “training” manipulations where working memory demands become progressively more difficult over time [1–3]. Even without an adaptive training design, working memory performance greatly improves over time with simple practice [4]. Here, we tested whether behavioral feedback aimed at reducing failures of visual working memory performance could augment simple practice benefits, and whether practice-related working memory benefits might lead to improvement on other cognitive tasks.

What are working memory failures, and why target them with feedback? Using a whole report working memory task, we can measure trial-by-trial fluctuations in working memory performance and identify failures [5,6]. In this task, participants briefly view an array of colored squares, remember them for a short duration, and then are required to report all items from the array. Accuracy for each trial is scored as the number of correctly reported items. By holding memory load constant at a difficult set-size (e.g. 6 items), we can measure endogenous fluctuations in performance. In this task, most participants have a mode of 3 items correct (and model fits are consistent with a mostly common capacity of 3 items), but they differ in how frequently they fail to reach this typical capacity limit. Surprisingly frequently (~12% of trials), participants perform no better than chance (0 or 1 correct). Other studies using change detection similarly have found that participants had lapses for a similar proportion of trials [7]. Mind wandering and attentional lapses occur frequently in everyday life [8–10], and lapses of attention influence all sorts of cognitive tasks, including working memory tasks. As such, reducing failures represents a potentially fruitful way to improve cognitive performance across a wide variety of domains. This is in part because of the simplicity of the goal. Rather than trying to boost maximal performance, we can simply try to eliminate abject failures.

The current study is related to, but distinct from, the broader “working memory training” literature. First, we focus specifically on practice benefits to a visual working memory task (which involves storage of color-space pairings) [5,6]. Some work has characterized training of visual working memory [11,12], but the vast majority of the working memory training literature has employed either the dual n-back task (which contains auditory and visual information and involves updating over time) or complex span tasks (which may contain verbal, numerical, or spatial information, and involve both “storage” and “processing” demands) [2,3,13,14]. Second, our experimental design compares working memory gains with practice alone versus practice with performance feedback. Here, we held task difficulty (set size) constant throughout practice. In contrast, the majority of the literature focuses on how well “adaptive” training designs may improve working memory performance (and whether these performance gains transfer to other domains).

Previous work has shown that visual working memory performance can be improved by giving participants trial-by-trial feedback about accuracy. The most effective working memory feedback focused participants on reducing failures rather than attempting to store more items [15]. In this feedback design, points were awarded for trials with at least 3 items correct, and points were subtracted for trials that did not reach this goal. Thus, this feedback structure used a weighted combination of both positive and negative feedback, and this weighted combination was more effective than positive feedback alone (e.g. +1 point per item). With weighted feedback, participants’ performance dramatically improved in blocks with feedback compared to without feedback. The improvement to working memory with weighted feedback is likely due to a combination of improved awareness of failures, motivation to reduce these failures [16], and changes to task strategy (for further discussion see [15]). Unfortunately, this improvement disappeared shortly after feedback was taken away. In a within-subjects design, participants who received feedback in the first half of the experiment experienced more failures immediately after the trial-by-trial feedback was taken away. Brief exposure to feedback may have been insufficient to produce lasting benefits on working memory performance.

Despite the relatively high frequency of working memory failures, participants are relatively unaware of them. Previously, we found that participants had poor meta-awareness of working memory failures, catching them only around 30% of the time [17]. Thus, we hypothesized that feedback may be effective in improving working memory performance because it helps to counter-act deficiencies in metaknowledge. Extensive exposure to external feedback may be necessary to bring about improvements in metaknowledge.

To test the potential for extensive feedback to provide lasting benefits to working memory performance, we had two groups of participants complete 6 practice sessions of a working memory task. One group completed the practice sessions with trial-by-trial feedback, the other group received no feedback. We also included an active control group (crossword puzzle practice) and a passive control group (no-contact) to measure baseline changes in working memory performance over time. We predicted that practicing with feedback would lead to stronger improvements in working memory performance, and that it would also lead to improvements in participant’s meta-awareness of working memory failures. We found robust effects of practice on working memory performance that were modestly augmented by the presence of feedback. However, we did not find evidence that feedback improves metaknowledge and we also found no “transfer” of working memory practice benefits to other cognitive tasks (visual search, antisaccade, Raven’s matrices).

Finally, we report the results of measures included to assess the validity of our design and participants’ expectations for improvement. First, we conducted post-hoc power analyses on current and previously published data, and we found that exposure to the task (i.e. practice effects) lowered power to detect a between-subjects effect. This decrease in power with practice may be important for planning new multi-session training studies with a between-groups effect. In addition, we included measures of participants’ effort, perceived improvement, and attitudes toward the malleability of intelligence. Subjective measures of effort and perceived improvement allowed us to test whether participants’ expectations were matched across training and control groups. We also included measures of subjects’ beliefs about intelligence (e.g. Theories of Intelligence Scale [18]) to test whether individuals’ beliefs predicted the degree of motivation- or placebo-related improvement from pre- to post-test (regardless of training group). For example, prior work by Jaeggi et al. [19] examining theories of intelligence [18,20] and working memory (n-back) training found that participants reporting more “fixed” theories of intelligence (e.g. “You can’t really change how intelligent you are”) showed less improvement from pre- to post-test, regardless of their training group (i.e. n-back versus active control).

## Materials and methods

### Participants

Procedures were approved by the University of Oregon Institutional Review Board. Participants were recruited from the University of Oregon and the surrounding community and provided written, informed consent. They were between the ages of 18 and 35 (*M* = 20.5, *SD* = 2.66), and they self-reported normal or corrected-to-normal visual acuity and normal color vision. Participants were paid a total of $200 for completing all sessions (2-hour pre-test, 2-hour post-test, and six 1-hour training sessions). They received $30 after the pre-test session. Upon completing all 6 training sessions and the post-test, they received an additional $170. If participants chose to withdraw from the study early, they were compensated at a pro-rated rate for their participation ($3.75 per 15 min). We initially recruited 79 subjects. A total of 6 participants withdrew after the pre-test, and 1 additional participant withdrew after the first training session. This left a total of 72 subjects (48 female) for analysis (23, 25, and 24 per group). At a later date, we recruited an additional 35 subjects to serve as a passive control group. A total of 2 participants no-showed for their first scheduled appointment, and an additional 4 participants did not return for their second appointment. This left 29 participants (22 female) for final analyses. These participants were paid $20 total for a 2-hour pre-test and $30 total for a 2-hour post-test.

### Procedures

Participants completed a 2-hour pre-test session, six 1-hour training sessions, and a 2-hour post-test session. After the pre-test, participants were pseudo-randomly assigned to one of three training groups. Pseudo-random assignment matched groups for average pre-test performance across all pre-test tasks (color whole report, crossword puzzles, color change detection, orientation whole report, visual search, antisaccade, Raven’s), as differences in pre-test performance often prevent sensible interpretation of results [13]. To do so, we randomly shuffled group assignment until all tasks yielded no main effect of Group (*p* >.05). Pseudo-randomization was performed once after the first 3 groups (“Working Memory—Feedback”, “Working Memory—No Feedback”, “Active Control”) completed the pre-test session but before any practice sessions occurred. The fourth group (“Passive Control”) was added at a later date and was not included in the pseudo-randomization procedure.

The critical training group (“Working Memory, Feedback”, n = 25) practiced the discrete whole report task and received feedback during their training sessions. A second group (“Working Memory, No Feedback”, n = 23) practiced the discrete whole report task but did not receive any feedback about their performance. A third group (“Active Control”, n = 24) practiced doing crossword puzzles. This active control group served as a baseline comparison to working memory practice while attempting to control for researcher contact and expectations for improvement [3,13]. Finally, a fourth group of subjects (“Passive Control, n = 29) completed only the pre-test and post-test sessions. Given concerns about the placebo effect causing differences in performance for passive control groups relative to active control groups, we wanted to include both.

During the pre-test and post-test sessions, participants completed six cognitive tasks in the same order (Color Change Detection, Color Whole Report, Orientation Whole Report, Visual Search, Antisaccade, Raven’s Advanced Progressive Matrices). During the post-test session, participants filled out some questionnaires after completing all the cognitive tasks. Cognitive tasks and questionnaires are described below. During training sessions, participants were required to practice their assigned task for the full hour.

All sessions took place in the lab in individual testing rooms. Crossword puzzles and questionnaires were completed with pencil and paper; all other cognitive tasks were administered on a PC running Windows XP with a 17-inch CRT monitor (refresh rate = 60 Hz). Stimuli were presented using MATLAB (The Mathworks, Natick, MA) with Psychtoolbox [21,22]. Participants were seated approximately 60 cm from the monitor.

### Training tasks

#### Color whole report

The color whole report task is a working memory task that measures trial-by-trial fluctuations in performance [5,6]. This task was used both for the Working Memory–Feedback group and for the Working Memory–No Feedback group. During each practice session, participants completed whole report trials for one hour in total; trials were divided into blocks of 30 trials each. The exact number of blocks completed varied across participants (*M* = 8.60, *SD* = .41). Critically, those in the Feedback group did not complete a different number of blocks per session compared to those in the No Feedback group (*p* = .41).

**Fig 1:**
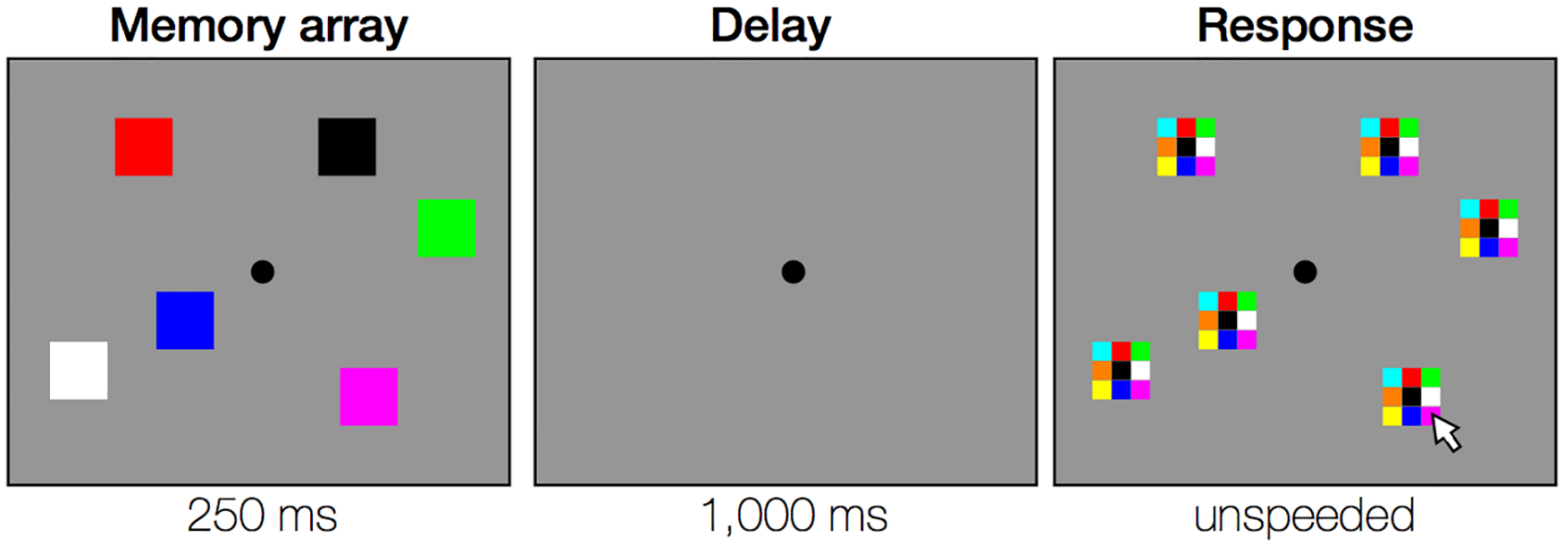
Trial procedures for the color whole report task. Key trial events are depicted from left to right. Participants remembered the locations and colors of 6 squares across a blank delay, then reported the color at each location by clicking a color in “response grids” with the mouse. The arrangement of colors in the response grids was the same for all trials and all participants.

On each trial (Fig 1), participants briefly viewed (250 ms) an array of 6 colored squares and remembered the items across a blank delay (1,000 ms). Colors were chosen from a set of nine easily discriminable colors (red, orange, yellow, green, blue, magenta, cyan, black, white). At test, place-holders containing a 3x3 grid of all possible colors appeared at the locations of the remembered items. The arrangement of colors in the response grid was fixed. Participants clicked the color in the place-holder corresponding to the color that was presented at each of the 6 locations. Accuracy for each trial was calculated as the number of correctly reported colors (out of 6 possible). After reporting colors for all items, the place-holders disappeared. The No Feedback group saw a blank gray screen after completing each trial. The Feedback group saw performance feedback after completing each trial. After presentation of feedback or a blank gray screen, the next trial began after a mouse click (followed by a 1,000 ms inter-trial interval). Performance feedback awarded participants points based on their performance. If participants performed well, they accrued points (+1 for 3 correct, +2 for 4 correct, +3 for 5 or 6 correct). If they performed poorly, they lost points or earned no additional points (-2 for 0 correct, -1 for 1 correct, 0 for 2 correct). In addition, participants earned a “streak bonus” for consistently performing well; the streak bonus was equal to the number of trials in a row that participants got at least 3 correct (e.g. 5 trials in a row = +5). Points accrued across each block of trials and reset to 0 at the beginning of each new block. Each trial’s feedback screen showed the number of correct items, the number of points gained or lost, total score for the current block, and an overall block high score. This points manipulation was previously shown to be very effective at reducing the frequency of working memory failures relative to no feedback or to simple feedback [15].

While making responses, participants were asked to indicate their confidence by using a mouse-button press [17]. If they were confident about an item, they were instructed to report the color of that item by clicking with the left mouse button. If they felt like they were guessing, they were instructed to instead use the right mouse button to respond. These confidence ratings can be used to calculate subjects’ metaknowledge of fluctuations in working memory performance.

#### Crossword puzzles

Participants were given a new packet of 3 crossword puzzles to work on during each 1-hour practice session. Puzzles were acquired from Boatload of Crosswords (Boatload Puzzles, Yorktown Heights, NY, USA).

### Pre- and post-test tasks

#### Color change detection

Change detection is a measure of working memory capacity [23]. On each trial, participants remembered an array of 4, 6, or 8 briefly presented (150 ms) colored squares across a blank delay (1,000 ms). Colors were chosen from a set of nine easily discriminable colors (red, orange, yellow, green, blue, magenta, cyan, orange, black, white). At test, a single test square was presented at the location of one of the remembered items. The color of the test square was the same or different from the remembered color (50% probability). If the color was the same, participants pressed the “z” key; if it was different they pressed the “/” key. Participants completed 5 blocks of 30 trials each.

#### Color whole report

Stimuli and trial procedures were the same as described for the No Feedback version of the training task. For the pre- and post-test assessments, participants completed 4 blocks of 25 trials each. Unless participants in the Feedback group can “transfer” feedback-related performance improvements to later task contexts (without feedback), then we would expect no main effect of feedback group during the post-test.

#### Orientation whole report

This task was very similar to the color whole report task. Instead of remembering color, participants instead remembered orientations of circles with wedges cut out from them (“wrench-head” stimuli). Orientations were chosen from one of 4 values (up, down, left, or right). On each trial, participants briefly viewed (200 ms) an array of 6 orientation stimuli and remembered the array across a blank delay (1,150 ms). At test, place-holders appeared at each of the remembered locations. These placeholders contained “cross-hairs” (intersected vertical and horizontal lines). Participants clicked the arm of the cross-hair that matched the remembered orientation. After participants responded to all items, the place-holders disappeared. The next trial began after a spacebar press (followed by a 500 ms inter-trial interval). Participants completed 2 blocks of 30 trials each.

#### Visual search

Visual search measures how quickly participants can find a target among distractors. Participants searched for an upright “L” among homogenous distractors (the letter “T” rotated 0, 90, 180, or 270 degrees). The vertical part of the target “L” was slightly offset so that it more closely matched the upside-down “T” distractors. Participants pressed the left arrow key if the slightly longer side of the “L” target was facing left, and they pressed the right arrow key if it was facing right. The number of distractors ranged from 1 to 8. There were 5 blocks of 48 trials.

#### Antisaccade

The antisaccade task is a measure of attention and cognitive control [24,25]. Participant fixated a cross in the center of the screen. After an unpredictable duration (.2–2.2 seconds), a cue (“=“) quickly flashed to the left or right of fixation. The cue flashed twice (100 ms on, 50 ms off, 100 ms on). Following a 50 ms delay, a target appeared in the opposite hemifield for 100 ms. The target was the letter “P”, “B”, or “R”. Following a 50 ms delay, the target letter was masked twice (letter “H” for 100 ms, blank 50 ms, number “8” for 100 ms). To detect the target, participants needed resist capture by the cue and quickly move their attention and eyes to the opposite hemifield. Participants reported the target by pressing the letter “P”, “B”, or “R” on the keyboard. For correct trials, the entire screen flashed green for 500 ms. For incorrect trials, the entire screen flashed red. There were 4 blocks of 36 trials total.

#### Raven’s Advanced Progressive Matrices

The Advanced Progressive Matrices task [26] is a measure of abstract reasoning ability and fluid intelligence. For each question, participants viewed a 3 by 3 grid of abstract geometric shapes. These shapes are related to one another (e.g. an abstract rule dictates similarity across columns / rows of the grid). The bottom right corner of the grid is missing, and participants must choose the item that best belongs from one of 8 choices. The full test is 36 questions that are presented in ascending order of difficulty. To measure change from pre- to post-test, questions were divided into two sets of 18 (even and odd questions). Most participants received even questions at pre-test and odd questions post-test (due to clerical error, 4 participants received odd questions at pre-test and even questions at post-test). Participants were given 10 minutes to work on the set of 18 questions; scores were calculated as the total number of correct questions.

#### Timed crossword puzzle

Participants were given 1 crossword puzzle, and they were given 10 minutes work on the puzzle. Participants were instructed that they should try to get as many words correct as possible in the allotted time. Crossword puzzle accuracy was scored as the total number of complete, correct words per minute of task time.

### Questionnaires

Questionnaires were administered at the end of the post-test session. All questions and scales administered to participants are available on our Open Science Framework page (https://osf.io/839dz/). We collected information about subject demographics (e.g. age, handedness). In addition, we were interested in whether participants thought that they improved from the pre-test to the post-test. We also used a few different questionnaires from Dweck (2000) to characterize the attitude of participants in our sample towards learning and intelligence. We reasoned that participants who thought they improved more or who view intelligence as more malleable might show greater practice benefits and/or placebo effects.

#### Demographic information and perceived performance

We collected participants’ age, gender, native language(s), handedness, and parental education level. We also asked participants to estimate their typical amount of sleep per night, and to give ratings of their average levels of alertness and motivation across the training sessions. In addition, we asked participants 6 questions about their perceived level of effort and improvement across their completed sessions. An example of subjects’ perceived effort is the statement, “I found it difficult to care very much about how I was doing on the tasks.” An example of subjects’ perceived performance is the statement, “I feel that my performance on the tasks improved from the first session to the last session.” Participants rated their endorsement of each statement from 1 (strongly agree) to 6 (strongly disagree). For ease of interpretation, these measures are plotted such that 6 represents the strongest agreement with each construct (e.g. more effort) rather than disagreement.

#### Theories of intelligence scale for adults

This 8-question scale [18] quantifies participants’ views on the malleability of intelligence. Participants rated their endorsement of each statement from 1 (strongly agree) to 6 (strongly disagree). People who view intelligence as more fixed (“entity theorists”) are more likely to endorse the statement, “To be honest, you can’t really change how intelligent you are.” Those who view intelligence as malleable (“incremental theorists”) would endorse a statement such as, “You can change even your basic intelligence level considerably.”

#### Goal choice items questionnaire

This 4-item questionnaire places learning goals against performance goals [18]. Learning goals represent engaging in an activity in which the participant would learn a lot, but would not necessarily perform well. Performance goals represent engaging in an activity where the participant would excel, but not necessarily be challenged [20]. For example, someone who values performance goals over learning goals would be more likely to endorse the statement, “Although I hate to admit it, I sometimes would rather do well in a class than learn a lot.” Previous work has shown that people who view intelligence as malleable are more likely to endorse learning goals over performance goals [27].

#### Confidence in one’s intelligence

This 3-item measure asks participants to rate their confidence in their intelligence [18]. This measure is most often used to show that those who view intelligence as fixed versus malleable do not differ in their confidence or optimism toward their own intelligence.

## Results

### Improvement on practiced task

To test whether feedback differentially changed the rate of improvement across all sessions, we ran a mixed ANOVA with between-subjects factor Feedback and within-subjects factor Session (Pre-test, 6 training sessions, and Post-test). We quantified behavioral performance in a couple of ways. First, we looked at mean performance (the average number of correct items on each trial). Second, we looked at the change of “poor performance” trials (less than 3 correct). The feedback manipulation used here incentivized participants to get 3 correct by awarding points only when this goal was achieved. Consistent with this incentive structure, we found previously that this feedback manipulation had the greatest impact on the proportion of poor performance trials [15]. As such, we expected *a priori* that this measure of performance should be most sensitive to any changes in performance.

**Fig 2:**
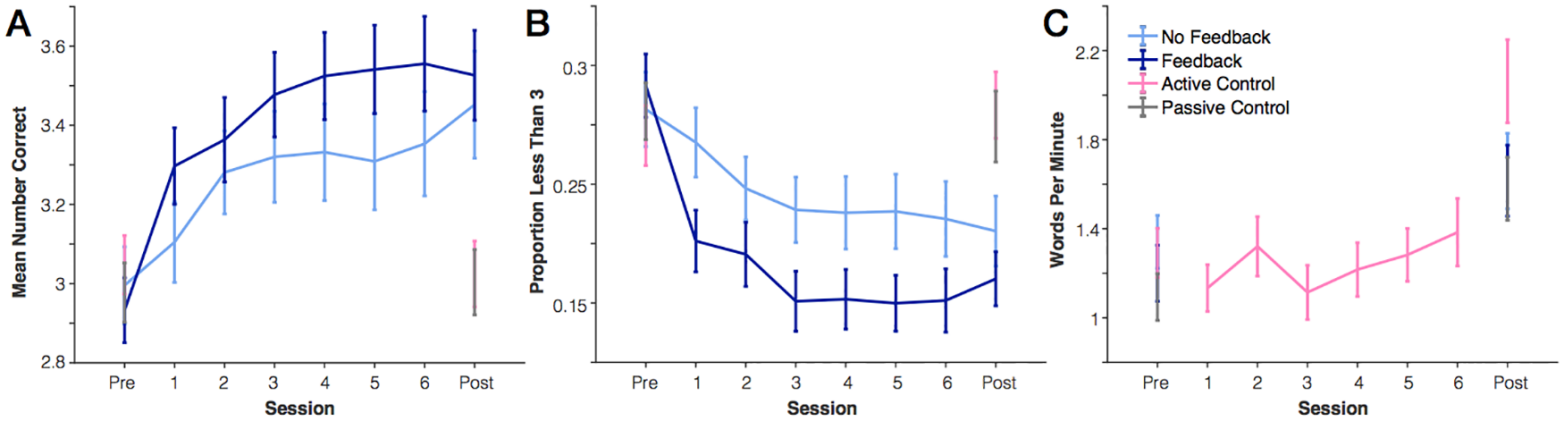
Improvement in practiced task over time. (A) Change in mean number correct for the working memory task. (B) Change in proportion less than 3 correct for the working memory task. (C) Change in crossword puzzle performance (words per minute). Error bars represent one standard error of the mean.

We found a large main effect of session on mean performance, *F*(2.52,115.71) = 30.0, *p* < .001, η_p_^2^ = .40, and proportion of poor performance trials, *F*(3.23,148.42) = 28.9, *p* < .001, η_p_^2^ = .39, indicating that practice led to significant improvements in working memory performance (Fig 2). Note, the Greenhouse-Geisser correction is applied where the assumption of sphericity is violated. There was no main effect of feedback for either measure (*p* > .11). However, there was a significant interaction between feedback and session for the poor-performance measure, *F*(3.23,148.42) = 3.68, *p* = .01, η_p_^2^ = .07, and a trending interaction for the mean performance measure, *F*(2.52,115.71) = 2.33, *p* = .09, η_p_^2^ = .05. Post-hoc comparisons revealed that the likely cause of the interaction was because of a larger difference between the feedback and no-feedback groups for earlier practice sessions. Two-tailed t-tests revealed a significant difference in the proportion of poor performance trials for only the first (*p* = .038) and third (*p* = .045) practice sessions. Indeed, ANOVAs testing for an interaction between pre-test scores and scores on the first session were significant (mean number correct, *p* = .004, proportion poor performance trials, *p* = .002). By design, there was no difference in pre-test scores between groups. Thus, this significant interaction supports the conclusion that there was an initial difference between feedback and no feedback groups in the first practice session that did not persist across all practice sessions.

**Fig 3:**
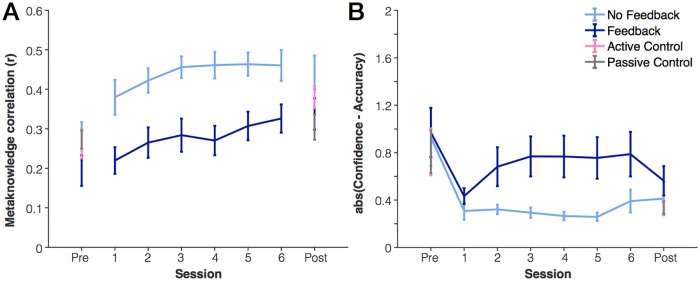
Improvement in working memory metaknowledge with practice. (A) Metaknowledge correlation metric. Higher correlation values indicate better metaknowledge. (B) Absolute value of the difference between confidence and accuracy on each trial. Values closer to 0 represent better metaknowledge. Error bars represent one standard error of the mean.

We also tested the hypothesis that participants in the feedback group would develop superior meta-knowledge relative to the feedback group (Fig 3). We found no supporting evidence for this hypothesis. To quantify metaknowledge accuracy that is relatively independent of overall bias in confidence reports, we calculated a “metaknowledge correlation” for each individual participant. To do so, we plotted the number of correctly reported items for each trial against the number of confident items for each trial then calculated the correlation coefficient (Pearson’s) for each participant. Note, some subjects occasionally used the same button (“no guess”) for all responses within the entire session. For these subjects, the correlation coefficient is undefined so they are excluded from the analysis (total remaining: 18 in no-feedback group, 20 in feedback group). We found that metaknowledge performance increased over time, as shown by increased correlation strength, *F*(3.91,140.86) = 6.58, *p* < .001, η_p_^2^ = .15. There was also a main effect of feedback, but not in the predicted direction. Those in the no-feedback group had higher metaknowledge than those in the predicted group, *F*(1,36) = 11.0, *p* = .002, η_p_^2^ = .24, and there was no interaction between feedback and session, *F*(3.91,140.86) = 1.4, *p* = .24, η_p_^2^ = .04. Post-hoc paired t-tests revealed that there were no significant differences between groups in either the pre-test (*p* = .27) or post-test sessions (*p* > .07). However, during all 6 training sessions the no-feedback group had higher metaknowledge relative to the feedback group (*p* < .001 to *p* = .015). This indicates that the main effect of group was dependent on the presence of feedback; the between-groups differences went away when no feedback was administered in the post-test session.

As a second test of metaknowledge that would not exclude any subjects, we instead looked at overall level of confidence relative to actual performance (measured as the absolute value of the difference between confidence and accuracy for each trial). Unlike the first measure, this difference measure is most similar to measures of “bias” in ratings, rather than accuracy. For this measure, values close to 0 would indicate better metaknowledge. This difference measure revealed the same significant effect of session; participants’ metaknowledge improved across sessions, *F*(3.07, 141.25) = 3.99, *p* = .009, η_p_^2^ = .08. There was again a main effect of feedback group, *F*(1,46) = 6.614, *p* = .013, η_p_^2^ = .13, and no feedback by session interaction (*p* = .32). Once again, the main effect of group was limited to sessions where feedback was actually presented. There was no significant difference between feedback groups for either the pre-test (*p* = .90) or post-test sessions (*p* = .42). However, there was a significant difference between groups for 4 of 6 practice sessions (*p* < .05). Unlike the change in metaknowledge correlation coefficient, the observed improvement over time was limited to the change from the pre-test to the first practice session. If only the 6 practice sessions were included in the ANOVA, there was no main effect of session (*p* = .29).

Across our two measures of metaknowledge, we found that practicing the working memory task led to improved metaknowledge (with or without feedback). However, this improvement was not necessarily acquired gradually over time; we found inconsistent time-courses of improvement for metaknowledge accuracy (correlation measure) versus bias (difference measure). Surprisingly, we also found that the presence of feedback altered metaknowledge in a counter-intuitive way. Specifically, those who received trial-by-trial feedback had *worse* metaknowledge relative to those who did not receive any feedback. However, these differences did not carry through to a post-test session with no feedback. This surprising finding may indicate that those who received feedback paid less attention to internal judgments.

Finally, we checked whether the active control group (crossword puzzles) improved on their practiced task over time (Fig 2C). A repeated-measures ANOVA with factor Session revealed a significant difference over time, *F*(3.57,78.56) = 24.52, *p* < .001, η_p_^2^ = .53. This difference was not entirely driven by the large rise from the last practice session to the final post-test. There was still a significant improvement across only the 6 practice sessions, *F*(5,110) = 4.67, *p* = .001, η_p_^2^ = .18, with a significant linear trend (*p* = .01). Thus, it seems that participants in the active control group were effortfully engaged throughout the practice sessions.

### Improvement from pre-test to post-test

**Fig 4:**
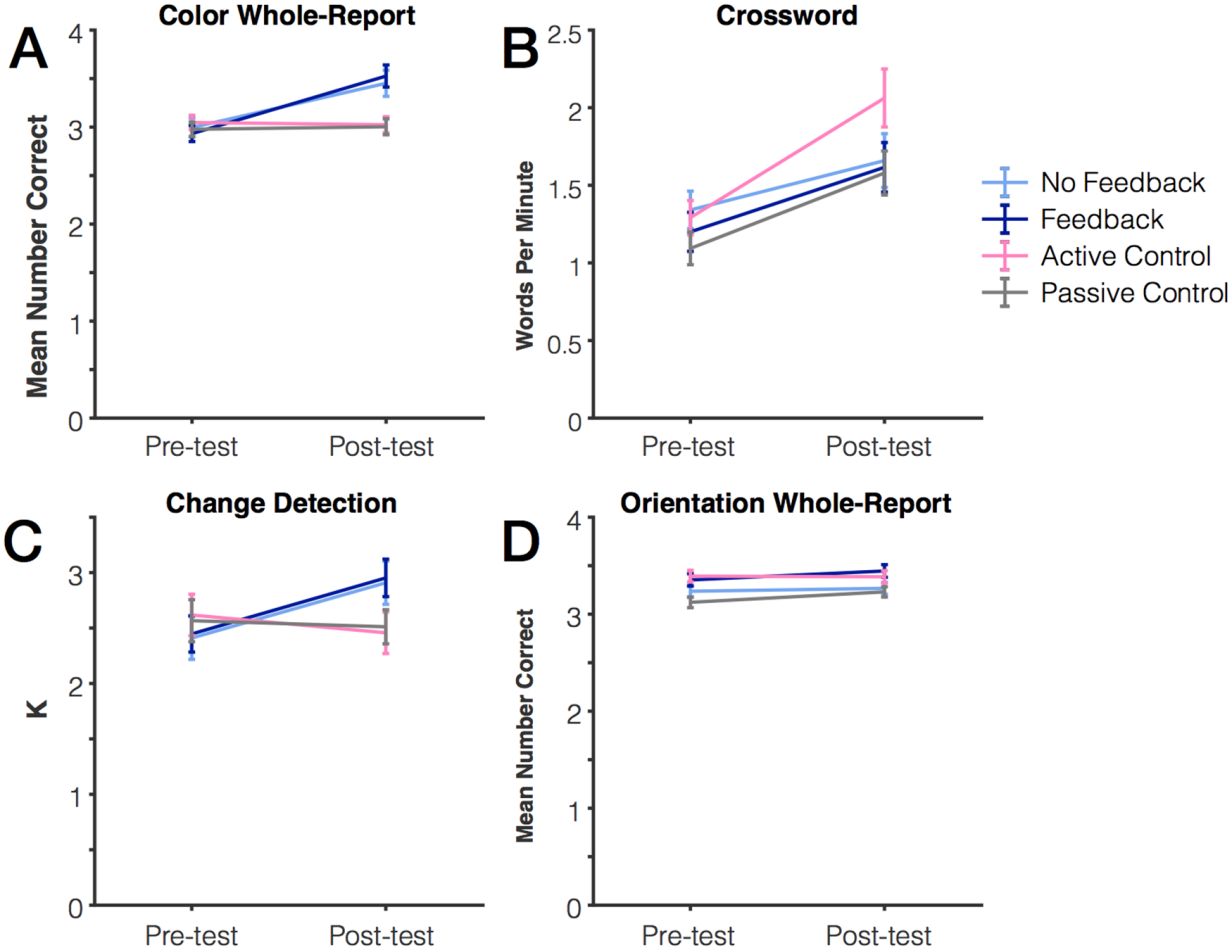
Change in performance from pre-test to post-test for practiced tasks and working memory measures. (A) Color whole report (B) Crossword puzzles (C) Color change detection (D) Orientation whole report.

First, we compared improvement from pre-test to post-test for each of the practiced tasks. We found that those who were in either of the two color whole report groups improved significantly more on the color whole report post-test measure relative to controls. Data for all four groups are plotted Fig 4. For this measure, there were no significant differences or between the two working memory groups (feedback vs. no feedback, *p* = .97) or the two control groups (passive vs. active, *p* = .67). As such, we collapsed the data into two groups: “working memory practice” and “control” groups. This analysis revealed a significant interaction between group and session, F(1,99) = 51.68, *p* < .001, η_p_^2^ = .34. Those who practiced the working memory task improved from pre- to post-test *t*(47) = 7.66, *p* < .001, whereas those in the control groups did not improve, *t*(52) = .13, *p* = .90. On the other hand, we did not find a significant interaction between these groups for improvement in metaknowledge performance (absolute value of number confident–number correct). Both groups improved their metaknowledge (*p* = .003), but there was no interaction between group and metaknowledge improvement (*p* = .21). However, this measure is difficult to interpret, as there were overall group differences at baseline (*p* = .02).

Likewise, participants in the crossword puzzle group improved more on the crossword puzzle post-test relative to the other three groups. Data for all four groups are plotted in Fig 4B. Accuracy for the crossword test was quantified as words per minute. One subject was excluded from the crossword puzzle analysis because of a clerical error (their test was not timed to precisely 10 minutes, and they were instead allowed an unknown extra amount of time to work). There was no difference in performance for any of the 3 groups who did not practice the crossword puzzle task (feedback, no feedback, and passive control), *p* = .68. As such, they were collapsed into one group and compared to the crossword puzzle group. Both the crossword puzzle practice group (*p* < .001) and the non-crossword group (*p* < .001) improved from pre- to post-test. However, the crossword group improved more than the non-crossword group as indicated by a significant interaction between group and session, F(1,98) = 8.78, *p* = .004, η_p_^2^ = .08.

Next, we examined whether practice on the color whole report working memory task led to improvements on the other two working memory tasks. These two tasks each differed in one aspect from the practiced task. The color change detection task used the same stimulus set (9 colors) but required a different response (same or different judgment). Conversely, the orientation whole report task used a different stimulus set (4 orientations), but used the same response mode (clicking each item).

We found evidence for stimulus-specific benefits of practice (Fig 4). Those in the working memory practice groups improved more on color change detection than did those in the control groups. There was again no difference between the two working memory groups (*p* = .86) or between the two control groups (*p* = .997) so they were collapsed. We found a significant interaction between group and session, F(1, 99) = 14.91, *p* < .001, η_p_^2^ = .13, indicating that those who received working memory practice improved more than did those in the control group. In fact, there was no significant change in the control group’s performance from pre- to post-test, *t*(52) = 1.09, *p* = .28. Unlike the color task, we did not find any evidence of a systematic benefit of color working memory practice on the orientation task. There were some baseline differences in performance across groups, so we did not collapse into two groups. We found a small benefit of practice on performance, F(1,51) = 6.03, *p* = .02, η_p_^2^ = .06, but no interaction between group and session, *p* = .24. We looked separately at each group for evidence of improvement from pre- to post- test, and we found that only the no-contact control group improved from pre- to post-test (*p* = .001). Thus, we found no evidence that practice with a color working memory task led to improvement on an orientation task. Instead, we conclude that the practice benefits obtained were highly feature-specific, similar to prior work examining visual working memory training [11] (but also see [19,28]).

**Fig 5:**
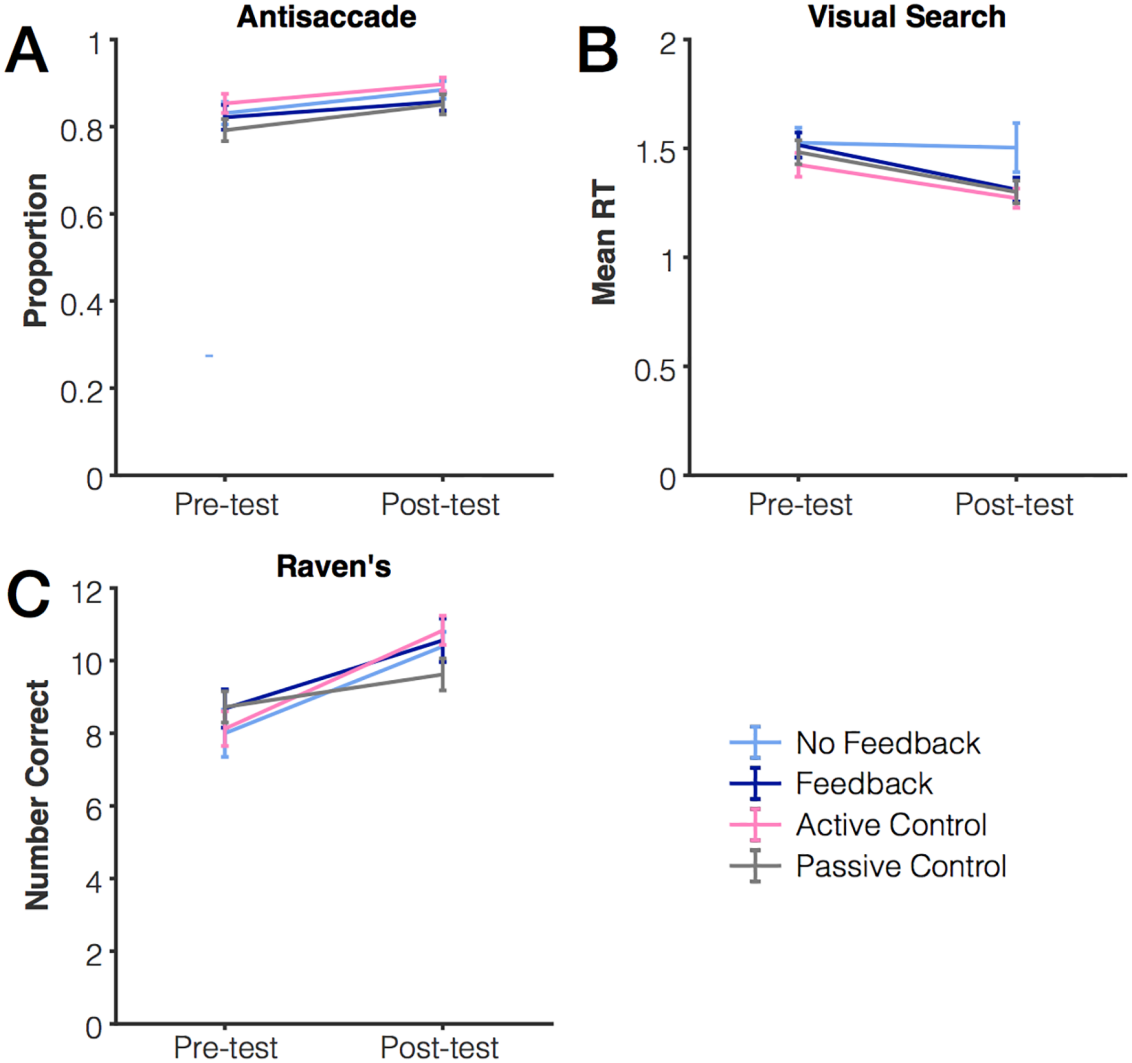
Change in performance from pre-test to post-test. (A) Antisaccade accuracy (B) Visual search average reaction time (C) Raven’s Matrices.

Finally, we found no differences in groups’ performance for any of the other cognitive tasks (Raven’s, Antisaccade, Visual Search), as shown in Fig 5. However, we did find overall improvement on these tasks from pre-test to post-test. Raven’s accuracy was scored as the total number of correct items completed in 10 minutes. There was an increase in accuracy on the Raven’s from pre- to post-test, F(1,97) = 48.47, *p* < .001, η_p_^2^ = .33, but no effect of group, F(3,97) = .29, *p* = .83, η_p_^2^ = .009, and no interaction between group and performance, F(3, 97) = 2.10, *p* = .11, η_p_^2^ = .06. Antisaccade performance was scored as percent error (wrong letter reported) and as average reaction time. There was an increase in antisaccade accuracy from pre- to post-test, F(1,97) = 23.84, *p* < .001, η_p_^2^ = .20, but no effect of group, F(3,97) = 1.27, *p* = .29, η_p_^2^ = .04, and no interaction between group and performance, F(3, 97) = .29, *p* = .84, η_p_^2^ = .01. The same pattern of results was seen for reaction time. Finally, visual search performance was quantified as the average response time. We found improvement in visual search reaction times from pre- to post-test, F(1,97) = 17.61, *p* < .001, η_p_^2^ = .15, but no effect of group, F(3,97) = 1.56, *p* = .20, η_p_^2^ = .05, and no interaction between group and performance, F(3, 97) = 1.41, *p* = .24, η_p_^2^ = .04. The same pattern of results was observed for search slopes. Thus, practicing a color working memory task did not lead to any marked improvement in other cognitive tasks.

Correlations between measures and the reliability of measures across pre- and post-test sessions are shown in S1–S3 Tables in the Supporting Information. Reliabilities for visual search reaction times and for Raven’s matrices were particularly poor (*r* < .5), so these tasks should be interpreted with caution. Reliability values for the working memory tasks were quite a bit higher for the control group (*r ~* .8) than for the working memory practice group (*r* ~ .6) presumably because there was some variability in how much participants’ benefited from practice (thus reducing the correlation between pre- and post-test).

### Growth mindset did not predict performance improvement

Next, we examined our hypothesis that participants with a growth mindset may show greater improvement relative to those with a fixed mindset. This effect might even bear out in tasks where we would expect no improvement due to practice alone. That is, those who think they are capable of growth might be more susceptible to expectancy-based placebo effects. We found no support for this hypothesis.

To create a single “growth mindset” score, we re-ordered reverse-scored items and then averaged across all 8 questions from the Theories of Intelligence scale (Cronbach’s alpha of the 8 items = .95). A score of 1 indicates the greatest possible degree of having a growth or “incremental mindset” whereas a score of 6 indicates the maximum degree of having a “fixed mindset”. The average growth mindset score was 2.72 (SD = 1.01, skew = .24), indicating that our sample leaned toward having a growth mindset (t-test compared to expected middle score of 3.5, *t*(100) = 7.82, *p* < .001, 95% CI [2.52, 2.92]).

For each of our cognitive measures, we computed a difference score (post-test–pre-test) as a measure of overall task improvement. We found no significant correlation between task improvement and growth mind-set for any of the tasks: Color Whole Report (*r* = -.01, *p* = .96), Crossword Puzzles, (*r* = .17, *p* = .09), Color Change Detection (*r* = .07, p = .47), Orientation Whole Report, (*r* = .05, *p* = .65), Raven’s (*r* = -.02, p = .82), Antisaccade accuracy (*r* = .02, *p* = .84), or Visual Search speed (*r* = -.04, p = .67). Likewise, there were no significant correlations between task performance and growth mindset when correlations were examined separately for control groups and working memory practice groups.

We also computed composite scores for the “goal choice” questionnaire and for the “confidence in intelligence” questionnaire. We found no relationship between the growth mind-set score and goal choice, *r* = .09, *p* = .39. We also found no relationship between growth mind-set and confidence in intelligence, *r* = .18, p = .08.

### Differences in perceived improvement and effort across groups

**Fig 6:**
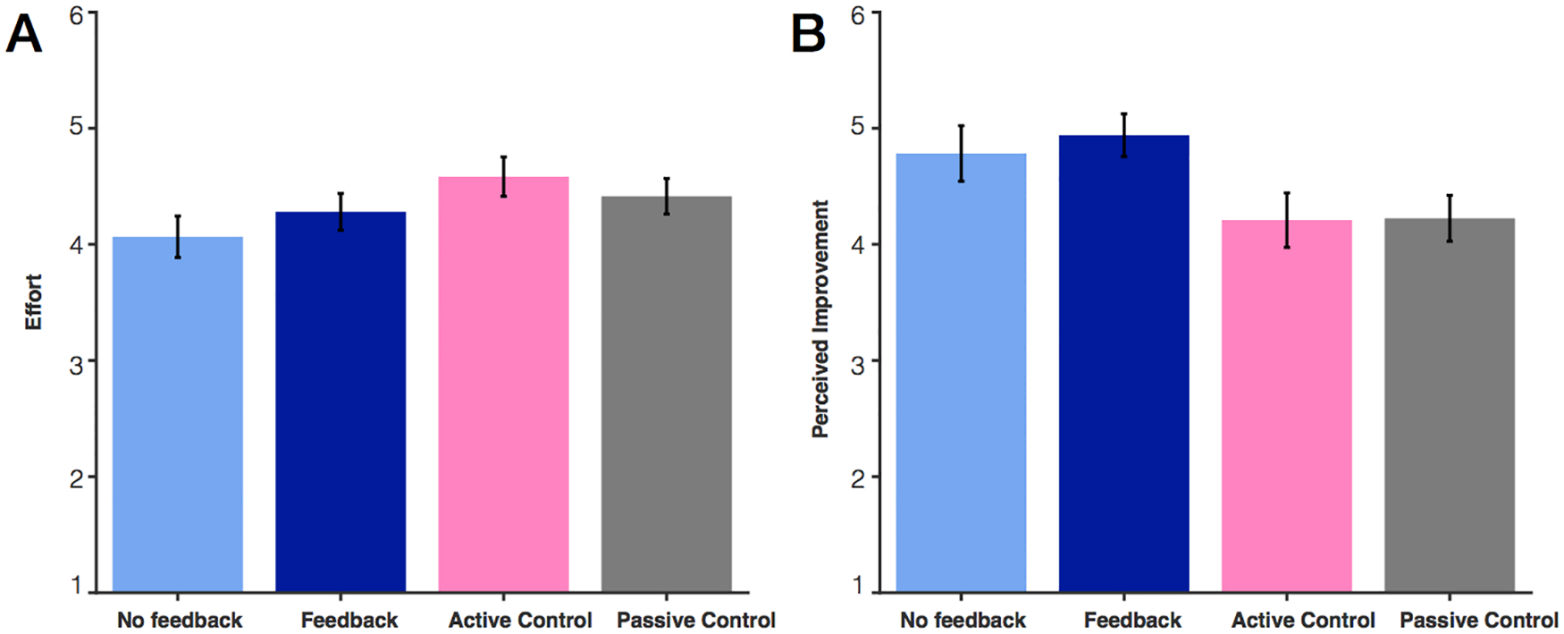
Difference in subjective effort and perceived improvement across groups. (A) Average subjective effort rating. (B) Average perceived improvement rating. Ratings were made on a scale from 1 to 6. In this plot, 6 represents the highest level of endorsement of effort and improvement.

We were interested whether those in different groups reported subjective differences in perceived improvement or effort (Fig 6). First, we looked at differences across all 4 groups using a one-way ANOVA. We found that there was no difference in self-reported effort between groups, F(3,97) = 1.67, *p* = .18. However, we did find a significant difference in perceived improvement, F(3, 97) = 3.12, *p* = .03. By eye, this effect of group appeared to be mostly driven by the difference between the two working memory groups relative to the controls. A post hoc test collapsing into two groups (“working memory task” or “control”), we found a difference in perceived improvement, F(1,99) = 9.37, *p* = .003. Although we found no large effect of subjective effort across the four groups, we nevertheless found differences in expectation (as indexed by subjective perceived improvement). This result emphasizes how difficult it is to find control conditions that can truly eradicate differences in expectation and control for placebo effects [29].

### Post-hoc power analyses based on prior work

We think it is important to qualify these results with a power analysis and a brief discussion of within- versus between-subjects manipulations in studies of working memory. In earlier work, we found robust within-subject effects of performance feedback on working memory performance [15]. However, because individual differences in working memory are both large and stable [4], the expected size of an intervention’s effect is typically much smaller than the range of individual differences. Unfortunately, we did not have the foresight to conduct both within- and between-subjects power analyses before collecting this experiment’s data. Post hoc, however, we can illustrate the extent of change to expected power for the same effect run between versus within subjects. Power estimates were calculated using the G*power 3.1 application [30,31].

First, we looked at the effect size and power for the feedback effect observed in Experiment 3 of Adam and Vogel (2016), as this was the same feedback manipulation used in the current study. In Experiment 3, participants received feedback for half of the experiment and no feedback for the other half of the experiment. The order of these two conditions was blocked and counterbalanced across participants. The within-subjects effect of feedback reported in Adam and Vogel (2016) was very strong, with a calculated effect size of *d* = .93 and power (1 − β) > .99 (n = 52). Next, we instead calculated effect size as between-subjects effects. We calculated between-subjects power separately for the first half and the second half of the experiment so that we could see if the computed effect size changed after subjects had exposure to the task (i.e. after experiencing one of the 2 conditions during the first half of the experiment). For the first half of the experiment, the between-subjects effect size was *d* = .78. With group sizes of 24 and 28, we found that power was sufficient, (1 − β) = .79. Note, these sample sizes are similar to the ones we chose for the present experiment. Unfortunately, however, the calculated effect size decreased in the second half of the Adam and Vogel (2016) experiment. The effect size for the second half of the experiment decreased to *d* = .51, (1 − β) = .44. Thus, had we run this full set of analyses before conducting the current experiment, we may have suspected that we might need larger group sizes to counteract a decrease in effect size potentially caused by practice effects or some other aspect of previous exposure to the task. For the first practice session in the present study (after exposure to the task in the pre-test), the difference between the feedback and no feedback groups was closer to the second, smaller effect found in Adam and Vogel (2016). We calculated an effect of size of *d* = .39, meaning that around 100 subjects per group would be needed to achieve power (1 − β) = .80 to detect a between-subjects effect within this single session. Given the robust effects of feedback for our earlier within-subjects manipulation and our relatively low power here, we speculate that the small effects of feedback that we saw would hold up with a larger between-groups sample. However, these results should nevertheless be interpreted conservatively; we see hints that feedback boosts practice benefits, but these effects are relatively short-lived. The relative effects of feedback appear to dissipate as practice effects increase.

## Discussion

Feedback can improve working memory performance when it points participants toward an optimal goal. Here, we asked whether practicing with feedback can help participants more quickly and efficaciously reduce the frequency of working memory failures. Our goal of reducing failures with feedback is similar to, but distinct from, the goal to increase capacity throughout the very large “working memory training” literature [1–3]. Both goals would have the same consequence of improving overall working memory performance but would rely upon distinct mechanisms and strategies. We hypothesized that practicing with feedback relative to without feedback would lead to faster, more robust improvements in working memory performance and would also improve subjects’ awareness of working memory failures. We found only partial support for these hypotheses. Consistent with our predictions, practicing with performance feedback increased working memory performance relative to practicing without feedback. However, the size of this effect was rather small and did not persist over time. Relative to the no-feedback group, participants in the feedback group more successfully reduced the frequency of poor performance trials only for some sessions. In addition, training with feedback did not improve subjects’ metaknowledge performance. If anything, subjects’ metaknowledge performance actually *declined* during feedback.

### The benefits of visual working memory practice are highly stimulus specific

Practicing on one visual working memory task led to improved performance on another visual working memory task with the same stimuli and a different response mode (color change detection) but led to no improvement on a task with different stimuli but the same response mode (orientation whole report). This finding is consistent with work by Gaspar and colleagues [11] showing that visual working memory training benefits were highly stimulus specific (extensive adaptive training on an object change detection task did not confer benefits to an orientation change detection task). Likewise, Buschkuehl and colleagues [12] recently found that adaptive visual working memory training yielded highly task-specific improvements. Future work is needed to establish the mechanisms underlying these stimulus-specific improvements to working memory performance (e.g. familiarity affecting encoding or storage [32], “chunking” strategies [33], or improved retrieval [34]). Unfortunately, the stimulus-specificity of visual working memory practice greatly limits the scope of the expected benefits of practice. Practice may be beneficial if the desired outcome is to more effectively remember or mentally manipulate the same stimulus set (e.g. the same set of icons in a complex software display), but may be of limited utility if transfer to novel stimulus sets is desired.

### Robust practice benefits with fixed task difficulty

Frequently, the use of an adaptive task is described as critical for yielding improvements to working memory performance [35], and direct comparisons have found that adaptive tasks yield larger improvements to performance than non-adaptive tasks [36–38]. However, when comparing adaptive and non-adaptive training protocols, it is important to control for difficulty, otherwise the manipulation of adaptiveness can be confounded with overall task difficulty during training. For example, work by von Bastian and Eschen [39] found that there was no difference between adaptive and non-adaptive working memory practice when overall difficulty was matched. Thus, mere exposure to a variety of difficulty levels has been shown to yield large increases in working memory performance.

In the current study, we found that exposure to a difficult condition alone was sufficient to yield robust increases in visual working memory performance. Participants never encountered any easy trials (all trials were set size 6), yet we observed robust improvements in performance across sessions. The effects of adaptive versus non-adaptive procedures have yet to be directly compared with a similar visual working memory task, so we cannot make strong claims about the efficacy of adaptive versus non-adaptive practice in this context. However, our finding of robust practice effects is consistent with previous observations of improvements to visual working memory performance across sessions, both for adaptive [12] and non-adaptive [4] tasks. An interesting open question for future work is to what extent participants may use “self-set” adaptive strategies when they are only given difficult trials, and whether such self-set goals underlie the practice benefits that we observed. Since a whole report task requires reporting each item individually, participants’ improvement may be aided by self-selected goals that change across practice, for example, “To start out, I’ll just try to get 2 correct and then guess on the rest.”

### Spacing may influence visual working memory practice benefits

Here, we reported robust improvements to visual working memory performance with practice, consistent with previous work [4,11,12]. However, inconsistent with this core result, a study by Olson and Jiang [40] reported that visual working memory is impervious to training. We think that the spacing of practice may explain the discrepancy between these two results. In Olson and Jiang (2004), all of the training was “massed” within a single practice session. Conversely, studies reporting robust practice effects on visual working memory performance have spaced practice across multiple days.

A “spacing effect” is defined by a larger performance benefit when an equivalent amount of practice is separated in time as opposed to being massed within a single practice session. Spacing effects have been extremely well-characterized in the domains of episodic memory [41,42] and motor learning [43], but have not been formally quantified in the working memory literature. However, a qualitative assessment of the working memory literature suggests that spacing effects in working memory may respect a non-monotonic function, like that reported for episodic memory [44,45]. With short “block breaks” within a single session (e.g., minutes), working memory practice effects are absent [40]. With intermediate spacing (e.g., 1 day– 1 week), a robust practice benefit is observed from the first to the second session [4,11,12]. However, with more distant spacing (e.g., many weeks to months), no robust practice benefit is observed (as for the Passive and Active Control Groups, Fig 4A; also see [46]). We think this is an intriguing qualitative pattern of results, but future work is needed to systematically quantify the effects of spaced practice on visual working memory performance.

### Previous exposure to tasks may reduce power

We found that practice decreased a between-groups effect size, which has profound implications for other studies attempting to calculate power for multi-session data based on single-session pilot studies. We reanalyzed an earlier dataset in which we could make the same comparison (feedback versus no-feedback) in both a within-subjects and a between-subjects manner. As expected, the overall between-subjects effect size was smaller than the within-subjects effect size. Critically, the same between-subjects effect size was reduced after all participants had experience with the task. This suggests that power estimates made from single-session task performance may not generalize well to multi-session studies. Thus, practice effects should be considered when planning sample sizes for large training studies.

### Feedback about accuracy did not improve metacognition

Consistent with previous work, we observed a reliable increase in metaknowledge performance with practice [47]. Surprisingly, however, we found that subjects’ metaknowledge performance was *worse* during sessions with feedback. This finding suggests that feedback may actually undermine the goal of improving metaknowledge if the feedback leads participants to spend less effort on monitoring their own performance. For example, if feedback emphasizes improving accuracy, then participants may neglect the secondary task of accurately rating their metaknowledge to better maximize available resources for the working memory task. Because our feedback focused on memory accuracy and did not reward metacognitive accuracy, we think that participants engaged in such a tradeoff.

Note, we have used the term “with feedback” to discuss behavioral effects related to our specific weighted feedback intervention relative to no feedback. However, we only tested a single feedback manipulation in this study, so our conclusions should not be interpreted to mean that all feedback manipulations would yield similar effects. Changes to the timing [48,49], frequency [50], content [15,16], or modality [16,51,52] of feedback would be expected to modulate the effect of feedback on behavioral performance. Manipulating the precise nature of performance feedback would be useful for investigating the limits of feedback-related improvements to working memory and metaknowledge performance. For example, a new weighted feedback design which combines both working memory accuracy and metacognitive accuracy may prove effective at boosting both working memory performance and metacognition. Finally, future work employing near real-time feedback about behavioral [53,54], neural [55–59], and physiological [60] markers of attentional state could be used to provide participants precise, theoretically-driven feedback and to test the specific mechanisms underlying feedback-related improvements.

### Mixed evidence that crossword puzzles are an adequate active control

One critical aspect of any intervention is the choice of a control group. The problem of placebo effects is particularly pernicious in multi-session behavioral interventions. Spurious placebo-like effects might be generated from differential amounts of experimenter contact, task engagement, or expectations for improvement [3,13,29]. If these issues cannot be avoided altogether, they can at least be measured. As an example, Boot and colleagues [29] performed an online study where they measured the expectations of participants. Participants viewed a training task (e.g. action video game) and then rated whether they thought by practicing that task they might improve on some other tasks and abilities. Unfortunately, Boot and colleagues found that expectations were not well matched by commonly-used control tasks (e.g. Tetris). Here, we used crossword puzzle practice as a control for working memory practice. Because people commonly believe that doing crossword puzzles reflects intelligence and can allow one to stay mentally “fit” [61,62], we thought this control task would have a good chance of leading subjects to believe that practice benefits were expected. Others, however, have speculated that crossword puzzles do not adequately control for demand and expectations relative to working memory tasks [63].

In our experiment, subjective measures of overall effort and perceived improvement (rated as average improvement for all pre- and post-test tasks) revealed mixed results for the efficacy of crossword puzzle control groups. On the one hand, those in the crossword puzzle group received the same amount of experimenter contact and did not report lower levels of effort throughout the experiment, indicating that they stayed engaged throughout the training sessions. On the other hand, they reported less perceived improvement than those in the working memory groups. Like the working memory groups, the crossword puzzle group did actually improve more on their trained task than the other groups. So, it would be accurate for this group to report that they improved *slightly* more (for the average across all of the tasks) than the passive control group. However, this was not the case. Instead, the active control group reported equivalent perceived improvement to the passive control group and lower perceived improvement than the two working memory practice groups. Thus, it is unclear whether or not crossword puzzle training adequately controlled for participant’s expectations relative to working memory practice groups, and future work is needed to more precisely characterize participants’ beliefs and expectations about a wide array of potential control tasks.

### Conclusions

We found robust practice-related improvements to visual working memory performance, both with and without performance feedback. Performance feedback somewhat augmented practice-related improvements, but the effects of feedback were somewhat weak and transient. Once subjects were well-practiced on the working memory task, the benefits of feedback dissipated. In addition, the benefits of feedback did not persist after feedback was taken away in the post-test session. Participants got better at monitoring their own performance with practice, but feedback did not play a role in this improvement. If anything, trial-by-trial feedback actually reduced participants’ self-monitoring and metacognitive accuracy. Finally, despite robust, stable improvements in working memory ability with practice, we found no evidence that practice benefits led to concomitant improvements in other cognitive abilities.

## Supporting information

S1 Table Correlations between pre-test measures. * p < .05 ** p < .01.
(DOCX)

S2 Table Correlations between post-test measures. * p < .05 ** p < .01.
(DOCX)

S3 Table Reliability of measures: Uncorrected correlation coefficient between pre- and post-test score. ^n.s.^ *p* > .05, all others *p* < .01.
(DOCX)
