# Supplementary material for: Improvements to visual working memory performance with practice and feedback
Source: PLoS One. 2018 Aug 30;13(8):e0203279. doi: 10.1371/journal.pone.0203279 (PMC6117037; doi:10.1371/journal.pone.0203279)
Supplement: S1 Table — * p < .05 ** p < .01. (DOCX) [file pone.0203279.s001.docx]

**S1 Table*.*** **Correlations between pre-test measures.**

|  | Color WR | Orientation WR | Change Detection | Antisaccade | Visual Search | Raven’s |
| --- | --- | --- | --- | --- | --- | --- |
| Color WR | - |  |  |  |  |  |
| Orientation WR | **.41**** | - |  |  |  |  |
| Change Detection | **.42**** | **.44**** | - |  |  |  |
| Antisaccade | **.26**** | **.26**** | .14 | - |  |  |
| Visual Search | -.17 | -.13 | -.20 | -.15 | - |  |
| Raven’s | .15 | .16 | .11 | **.21*** | -.02 | - |
| Crossword | **.27**** | .15 | .02 | **.26**** | **-.25*** | .07 |

* p < .05 ** p < .01
