# Supplementary material for: Improvements to visual working memory performance with practice and feedback
Source: PLoS One. 2018 Aug 30;13(8):e0203279. doi: 10.1371/journal.pone.0203279 (PMC6117037; doi:10.1371/journal.pone.0203279)
Supplement: S2 Table — * p < .05 ** p < .01. (DOCX) [file pone.0203279.s002.docx]

**S2 Table*.*** **Correlations between post-test measures.**

|  | Color WR | Orientation WR | Change Detection | Antisaccade | Visual Search | Raven’s |
| --- | --- | --- | --- | --- | --- | --- |
| Color WR | - |  |  |  |  |  |
| Orientation WR | **.51**** | - |  |  |  |  |
| Change Detection | **.56**** | **.30**** | - |  |  |  |
| Antisaccade | **.29**** | **.33**** | .18 | - |  |  |
| Visual Search | .09 | -.09 | .15 | **-.29**** | - |  |
| Raven’s | **.31**** | **.32**** | .18 | .15 | .03 | - |
| Crossword | **.25*** | **.25*** | .04 | **.25*** | -.18 | .14 |

* p < .05 ** p < .01
