# Supplementary material for: Improvements to visual working memory performance with practice and feedback
Source: PLoS One. 2018 Aug 30;13(8):e0203279. doi: 10.1371/journal.pone.0203279 (PMC6117037; doi:10.1371/journal.pone.0203279)
Supplement: S3 Table — n.s. p > .05, all others p < .01. (DOCX) [file pone.0203279.s003.docx]

**S3 Table. Reliability of measures: Uncorrected correlation coefficient between pre- and post-test score.**

|  | All Subjects | Working Memory Groups | Control Groups |
| --- | --- | --- | --- |
| Color WR | .62 | .62 | .86 |
| Orientation WR | .74 | .67 | .80 |
| Change Detection | .57 | .49 | .72 |
| Antisaccade | .67 | .50 | .81 |
| Visual Search | .46 | .28^n.s.^ | .77 |
| Raven’s | .33 | .45 | .19^n.s.^ |
| Crossword | .77 | .81 | .76 |

^n.s.^ *p* > .05, all others *p* < .01
